# CD47;Rag2;IL-2rγ triple knock-out mice pre-conditioning with busulfan could be a novel platform for generating hematopoietic stem cells engrafted humanized mice

**DOI:** 10.3389/fimmu.2024.1365946

**Published:** 2024-07-26

**Authors:** Kang-Hyun Kim, Sang-wook Lee, In-Jeoung Baek, Hye-Young Song, Seon-Ju Jo, Je-Won Ryu, Seung-Hee Ryu, Jin-Hee Seo, Jong-Choon Kim, Seung-Ho Heo

**Affiliations:** ^1^ Convergence Medicine Research Center, Asan Medical Center, Seoul, Republic of Korea; ^2^ College of Veterinary Medicine, Chonnam National University, Gwangju, Republic of Korea; ^3^ Department of Radiation Oncology, Asan Medical Center, Seoul, Republic of Korea; ^4^ Asan Institute for Lifesciences, Asan Medical Center, Seoul, Republic of Korea; ^5^ Korea Radioisotope Center for Pharmaceuticals, Korea Institute of Radiological and Medical Sciences, Seoul, Republic of Korea

**Keywords:** busulfan, cd47, hematopoietic stem cells, humanized mice, signal-regulatory protein alpha, total body irradiation

## Abstract

**Introduction:**

Humanized mouse models to recapitulate human biological systems still have limitations, such as the onset of lethal graft-versus-host disease (GvHD), a variable success rate, and the low accessibility of total body irradiation (TBI). Recently, mice modified with the CD47-SIRPA axis have been studied to improve humanized mouse models. However, such trials have been rarely applied in NOD mice. In this study, we created a novel mouse strain, NOD-CD47^null^Rag2^null^IL-2rγ^null^ (RTKO) mice, and applied it to generate humanized mice.

**Methods:**

Four-week-old female NOD-Rag2^null^IL-2rγ^null^ (RID) and RTKO mice pre-conditioned with TBI or busulfan (BSF) injection were used for generating human CD34+ hematopoietic stem cell (HSC) engrafted humanized mice. Clinical signs were observed twice a week, and body weight was measured once a week. Flow cytometry for human leukocyte antigens was performed at intervals of four weeks or two weeks, and mice were sacrificed at 48 weeks after HSC injection.

**Results:**

For a long period from 16 to 40 weeks post transplantation, the percentage of hCD45 was mostly maintained above 25% in all groups, and it was sustained the longest and highest in the RTKO BSF group. Reconstruction of human leukocytes, including hCD3, was also most prominent in the RTKO BSF group. Only two mice died before 40 weeks post transplantation in all groups, and there were no life-threatening GvHD lesions except in the dead mice. The occurrence of GvHD has been identified as mainly due to human T cells infiltrating tissues and their related cytokines.

**Discussion:**

Humanized mouse models under all conditions applied in this study are considered suitable models for long-term experiments based on the improvement of human leukocytes reconstruction and the stable animal health. Especially, RTKO mice pretreated with BSF are expected to be a valuable platform not only for generating humanized mice but also for various immune research fields.

## Introduction

In various fields of biomedical research, disease models using immunocompetent mice are beneficial and widely used tools. However, there are limitations in recapitulating human biological systems using mouse models because of the genetic and immunological differences between mice and humans, especially in studies involving the human immune response (1). To address these limitations, humanized mouse models, which are engrafted with human hematopoietic cells or lymphoid tissues in immunodeficient mice, have been developed (1, 2).

Humanized mouse models can be classified into the following three categories; human peripheral blood lymphocytes (hPBL), human CD34+ hematopoietic stem cells (hCD34+), and human bone marrow-liver-thymus (BLT) engrafted models (2, 3). The hPBL model, which is generated by intravenous or intraperitoneal injection of human peripheral blood mononuclear cells (PBMC) into immunodeficient mice, is considered the fastest, simplest, and most economic model. However, there is a narrow experimental window (4~6 weeks after PBMC injection) because of the occurrence of lethal graft-versus-host disease (GvHD), and this is the main limitation of the model (4, 5). The BLT model generated by implantation of human fetal liver and thymus tissues into immunodeficient mice provides the microenvironment of the human thymus and represents a complete human immune system, including the development of human T cells. However, the availability of human tissues is seriously restricted, and GvHD is more severe in the BLT model than the hCD34+ model due to T cell affinity for the mouse major histocompatibility complex (3, 4, 6). The hCD34+ model is created by the injection of human CD34+ hematopoietic stem cells (HSCs) after total body irradiation (TBI) or the administration of myelosuppressive agents such as busulfan (BSF). This model has advantages, including the immune reconstitution of all human hematopoietic lineages, a low incidence of GvHD as compared to other models, and a relatively long-term experimental window. On the other hand, the relatively long period for humanization of more than 10 weeks, low accessibility of TBI, and a variable success rate in humanization are disadvantages of this model (3, 4). To overcome the limitations of each humanized mouse model, various research trials such as additional human cytokine treatments, modifications of human cells, and the application of new genetically engineered mice strains have been performed (1, 4).

Numerous immunodeficient mouse strains are applied for generating humanized mouse models, and NOD-scid IL2rγ^null^, NOD-Rag1/2^null^IL2rγ^null^, Balb/c-Rag2^null^IL2rγ^null^, and related strains are representative models (7). The signal-regulatory protein alpha (SIRPA) gene of the NOD mouse strain has a strong affinity for human CD47, which could facilitate engraftment of human leukocytes (8). The Rag1/2 mutation has the advantage of irradiation tolerance and no occurrence of T/B cell leakiness (9). Therefore, the NOD-Rag1/2^null^IL2rγ^null^ strain is a more suitable platform for generating the hCD34+ model than the NOD-scid IL2rγ^null^ strain.

SIRPA, also known as CD172a, is an immunoglobulin superfamily protein that is abundantly expressed mainly on macrophages and myeloid cells. CD47, also called integrin-associated protein, is expressed ubiquitously, including on leukocytes, and it interacts with SIRPA. The interaction of CD47 and SIRPA plays a role in inhibiting host cell phagocytosis. Therefore, CD47 is functionally known as a “don’t-eat-me” signal (10). Furthermore, CD47 of the SIRPA signaling system plays an important role in the engraftment of human tissues and HSCs (11). Recent studies of humanized mouse models using CD47 knockout immunodeficient mice demonstrated enhanced engraftment of human cells and reduced GvHD occurrence (12–15). However, there are few studies about CD47 gene modification using NOD background mice (8).

In this study, we generated the hCD34+ model using NOD-Rag2^null^IL2rγ^null^ (RID) and NOD-CD47^null^Rag2^null^IL2rγ^null^ (RTKO) mice, and their immunological and pathological features were examined in detail. RID mice are suitable for generating hCD34+ models (9), and we expected that human cell engraftment and GvHD occurrence would be improved in RTKO mice. At the same time, two pre-conditioning methods, TBI and BSF injection, were compared to improve on the low accessibility of TBI due to the cost of the irradiator and strict regulations (16), and to evaluate whether BSF injection is also effective in the RTKO mouse, a new genetically engineered strain.

## Materials and methods

### Animals

**Figure 1:**
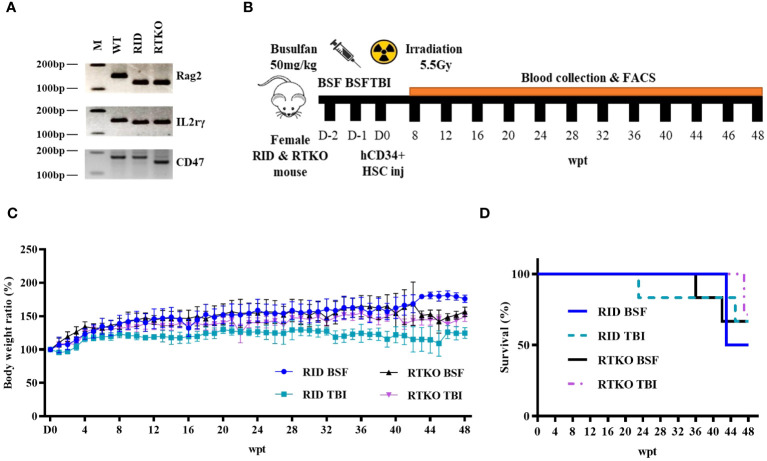
Genotyping, scheme, and clinical observation of the hematopoietic stem cell engrafted humanized mouse model. **(A)** The representative PCR results of wild type, RID, and RTKO mouse. **(B)** Scheme for the generation of the humanized mouse. Four-week-old female RTKO and RID mice were used to generate humanized mice. For FACS analysis to confirm human leukocyte engraftment, peripheral blood was collected by retro-orbital bleeding from mice at four-week intervals from eight to 44 weeks and at two-week intervals from 44 to 48 weeks after hCD34+ HSC injection. **(C)** The body weight ratio of humanized mice (presented as percentage of original body weight determined at the time of HSC injection) and **(D)** the survival rate changes were monitored weekly after HSC injection. The number of mice in each experimental group was as follows; RID BSF n=6, RID TBI n=6, RTKO BSF n=6, RTKO TBI n=7; M, a ladder marker; WT, wild type; RID, Rag2; IL-2rγ double KO NOD mice; RTKO, CD47; Rag2; IL-2rγ triple KO NOD mice; FACS, fluorescence-activated cell sorting; HSC, hematopoietic stem cell; BSF, busulfan; TBI, total body irradiation; wpt, weeks post transplantation.

All procedures were approved by the Institutional Animal Care and Use Committee of the Asan Institute for Life Sciences (Seoul, Korea, IACUC No 2020-12-093). CD47 KO NOD mice were kindly provided by Dr. In-Jeoung Baek (Convergence Medicine Research Center, Seoul, Korea), and Rag2;IL2rγ double KO NOD (RID) mice were obtained from GEM Biosciences (Cheongju, Korea). CD47;Rag2;IL2rγ triple KO NOD (RTKO) mice were generated by mating CD47 KO NOD mice and RID mice. Genotyping for the RTKO mice was examined by polymerase chain reaction (PCR) using the Taq polymerase (ebt 1201; Elpis bio, Daejeon, Korea) and TAKARA PCR Thermal Cycler Dice (TP600; Takara, Tokyo, Japan). The primer information is listed in **Table 1**. The amplified DNA was evaluated by electrophoresis using 4% agarose (Lonza, Rockland, ME, USA) gel, and subsequent ethidium bromide (ER2003-020-00; Biosesang, Yongin, Korea) staining. Gel imaging was obtained using the Gel Doc XR+ Gel Documentation System (Biorad, Hercules, CA, USA) (**Figure 1A**). All mice were maintained in the laboratory animal breeding room under specific pathogen-free conditions.

**Table 1:** Sequences of polymerase chain reaction primers used to genotype the knockout alleles of the Rag2, IL2rγ, and CD47 genes.

Gene	Sense	Sequence	Product size KO (WT)	Tm (°C)
Rag2	Forward Reverse	5′-TGT CTG TCG CTT GCA AGA AT-3′ 5′-CCA AAG AGA ACA CCC ATG CT-3′	128 bp (142 bp)	62.9
IL2rγ	Forward Reverse	5′-TAC TCT GCC CCT TCC AGA GG-3′ 5′-CTT CTT CCC GTG CTA CCC TC-3′	134 bp (144 bp)	68
CD47	Nested 1^st^ Forward Nested 1^st^ Reverse Nested 2^nd^ Forward Nested 2^nd^ Reverse	5′-GAC ACG AAG CCG GAA GAG AG-3′ 5′-TGC GGT TGT TCC CAG TTC TT-3′ 5′-GTT TCC CTT GAA GGC AGC AG-3′ 5′-GGC GCC TGG GTG CTG-3′	671 bp (697 bp) 148 bp (174 bp)	68 62.9

### Experimental design

4-week-old female RID and RTKO mice were utilized for generating HSCs engrafted humanized mice following myelosuppression. Two myelosuppressive methods were applied for immune suppression: TBI with 550 cGy (X-rad 320; Precision x-ray irradiation, Madison, CT, USA) or intra-peritoneal injection of 50 mg/kg BSF (Otsuka Pharmaceutical Co. Ltd., Tokyo, Japan). BSF was administered twice at an interval of 24 hours at a dose of 25 mg/kg. For humanization, hCD34+ HSCs were purchased from Lonza (2C-101; Basel, Switzerland), and HSCs were cultured with RPMI-1640 medium (Thermo Fisher Scientific, Waltham, MA, USA). After one day of incubation, 1X10^5^ HSCs suspended in phosphate-buffered saline (200 µL) were intravenously injected within 24 hours after myelosuppression (**Figure 1B**). Enrofloxacin (0.27 mg/mL; Bayer, Leverkusen, Germany) was added to the drinking water to prevent bacterial infection (16). Clinical signs were observed twice a week, and body weight was measured once a week. After inhalation anesthesia using 2% isoflurane (Terrel™, Piramal Critical Care, Inc., Bethlehem, PA, USA), 100*μ*l of blood from the retro-orbital plexus was collected every four weeks from eight to 44 weeks and every two weeks from 44 to 48 weeks after the hCD34+ HSC injection. Mice were sacrificed at 48 weeks after the HSC injection. At the time of the sacrifice, 50mg/kg of alfaxan (Jurox, Rutherford, Australia) and 10mg/kg of xylazine (Rompun™; Elanco, Ansan, Korea) were injected via intraperitoneal route for general anesthesia (17, 18). After confirming that mice were fully anesthetized, they were then sacrificed by exsanguination from the inferior vena cava for the further analysis.

### Flow cytometry analysis

In peripheral blood samples taken from the mice, red blood cells were lysed by RBC lysis buffer (Biolegend, San Diego, CA, USA) and then stained with eight types of fluorescein-conjugated antibodies. The following antibodies specific for human antigens were employed: anti-hCD45-pacific blue (HI30), anti-hCD3-APC-Cy7 (SK7), anti-hCD4-FITC (RPA-T4), anti-hCD8-PE (RPA-P8), anti-hCD19-PerCP-cy5.5 (HIB19), and anti-hCD56-Amcyan (NCAM16.2) from Becton Dickinson Biosciences (Franklin Lakes, NJ, USA); anti-hCD14-APC (HCD14) and anti-hCD66b-PE-Cy7 (G10F5) from Biolegend. Data were acquired on a FACS Canto II Flow cytometer (BD Biosciences) and analyzed using FACSDiva 8.0.2 (BD Biosciences).

### Hematoxylin and eosin staining and histopathological analysis

Fixed lung, liver, kidney, and skin tissues were processed by standard methods, embedded in paraffin, and then cut into 4-μm sections. The sections were deparaffinized, rehydrated, and stained with H&E. The sections were then dehydrated, cleared, mounted, and viewed by light microscopy. A semi-quantitative scoring system (0 to 5 grades), based on the severity of the lesion, was applied for the histological assessments as follows (19, 20): (0) Normal; (1) Minimal: minimal inflammatory cell aggregation; (2) Mild: inflammatory cell aggregation (≤ 10%), minimal apoptosis or necrosis, epithelial thickening (≤ 30 µm); (3) Moderate: inflammatory cell aggregation (≤ 25%), apoptosis or necrosis, epithelial thickening (≤ 60 µm); (4) Severe: inflammatory cell aggregation (≤ 50%), necrotic foci, epithelial thickening (≤ 100 µm); (5) Generalized: inflammatory cell aggregation (≥ 50%), necrotic foci, epithelial thickening (≥ 100 µm). Two veterinary pathologists independently reviewed all of the lesions.

### Immunohistochemical staining

For immunohistochemistry, selected serial sections (4 μm) were deparaffinized, rehydrated, placed in 0.01 M citrate buffer (pH 6.0), and heated in a microwave for 15 min. Then, the slides were incubated for 10 min in 1.0% H2O2. The slides were preincubated with blocking serum (Vectastain ABC kit; Vector Laboratories, Burlingame, CA, USA), incubated with rat anti-human CD45 (MA5-17687, 1:100, Invitrogen), rabbit anti-human CD3 antibodies (PA5-32318; 1:1,000, Invitrogen), and mouse anti-human CD19 (14-0199-82; 1:50, Invitrogen). The sections were incubated with biotinylated secondary antibodies followed by incubation with avidin-coupled peroxidase (Vectastain ABC kit; Vector Laboratories). The CD19 antibody was stained using the Mouse on Mouse (M.O.M.) detection kit (Vector Laboratories). After development with 3,3’-diaminobenzidine (DAB Substrate kit, Vector Laboratories), the slides were counterstained with hematoxylin.

### Western blot analysis

Protein was extracted from spleen tissue with extraction solution (Pro-PrepTM; Intron Biotechnology, Seoul, Korea). The protein concentrations were determined using a BCA kit (Pierce Biotechnology Inc., Rockford, IL, USA). After being electrophoresed on SDS-PAGE and transferred onto nitrocellulose membranes, the proteins were blocked and incubated with specific antibodies against anti-mouse β-actin (A5441; 1:5000, Sigma-Aldrich, St. Louis, MO, USA), anti-human CD3 (PA5-32318; 1:50, Invitrogen), anti-human CD19 (PA5-11578; 1:1000, Invitrogen), anti-human CD45 (MA5-15478; 1:500, Invitrogen) at 4˚C. Then, the membranes were washed with Tris-buffered saline with Tween^®^ 20 detergent (GIM003; Dongin biotech Biotech, Seoul, Korea) and incubated with either anti-rabbit or anti−mouse secondary antibodies (Jackson ImmunoResearch, West Grove, PA, USA), which were horseradish-peroxidase linked. Specific antibodies were detected with an ECL test kit (Kirkegaard & Perry Laboratories Inc., Gaithersburg, MD, USA). The band intensities were quantified using Imagequant Software (Image Lab V4.0; Bio-Rad Inc., San Diego, CA, USA) and normalized to β-actin expression.

### Multiplex human cytokine analysis

At the time of sacrifice, whole blood of each mouse was collected from the inferior vena cava, and the serum was used for measuring human cytokines with a Human CorPlexTM Cytokine Panel 1 10-Plex Array (116-7BF-1-AB; Quanterix, Billerica, MA, USA) according to the manufacturer’s instructions. The array measured cytokine concentrations of human IL-1β, IL-4, IL-5, IL-6, IL-8, IL-10, IL-12p70, IL-22, IFN-γ, and TNF-α. The results were acquired and analyzed with the SP-X Imaging System (Quanterix).

### Statistical analysis

For statistical analysis, all data obtained were analyzed using SPSS V14.0 software (SPSS Inc., Chicago, IL, USA). Statistically significant differences between the studied groups were evaluated using the unpaired Student’s t-test or Fisher’s exact test. Results were determined to be statistically significant for values of p<0.05 (p<0.05 and p<0.01 are indicated in the Figure legends).

## Results

### Generation of humanized mice and clinical signs

Four-week-old female RID and RTKO mice were employed for generating humanized mice following myeloablation and transplantation of HSCs. The mice were euthanized 48 weeks after HSCs administration (**Figure 1B**). To determine the animal health status and the severity of GvHD, the mice were weighed once a week. The body weight (BW) ratio increased the most in the RID BSF group and the least in the RID TBI group. The BW ratio of the RID BSF group was significantly higher during most of the period as compared to the RID TBI group from nine weeks post transplantation (wpt) and the RTKO group from 43 wpt. The BW ratio of the RTKO BSF group was greater than that of the RTKO TBI group; however, there was no meaningful difference (**Figure 1C**). Clinical symptoms such as hyperkeratosis, hair loss, cachexia, anemia, and jaundice were observed, and some mice died during the experiments. In the RID BSF group, three mice died at 43 wpt, and in the RID TBI group, one mouse died each at 23 and 45 wpt, respectively. In the RTKO BSF group, one mouse died each at 36 and 42 wpt, and two mice died at 47 wpt in the RTKO TBI group. Overall, the 48 wpt survival rate was the highest in the RTKO TBI group (71.4, n=7), and the lowest in the RID BSF group (50.0%, n=6), while the survival rates of the RID-TBI group and the RTKO BSF group were each 66.7% (n=6) (**Figure 1D**). The detailed information on animal mortality is described in **Table 2**.

**Table 2:** The detailed information on animal mortality.

Groups	Number and timing of deaths	Clinical signs or necropsy findings
RID BSF group	One dead mouse at 43 wpt Two dead mice at 43 wpt	Sudden death, no weight loss, moderate alopecia and hyperkeratosis. The breeding management problem (a water bottle leak and starvation)
RID TBI group	One dead mouse at 23 wpt One euthanized mouse at 43 wpt	Sudden death, no weight loss, mild alopecia and hyperkeratosis, moderate splenomegaly. Weight loss 16.5%, jaundice, hunched posture, reduced activity.
RTKO BSF group	One dead mouse at 36 wpt One euthanized mouse at 42 wpt	Sudden death, no weight loss, moderate hepatic inflammation. Weight loss 12.5%, anemia, hunched posture, reduced activity.
RTKO TBI group	One euthanized mouse at 47 wpt One euthanized mouse at 47 wpt	Weight loss 8.1%, anemia, hunched posture, reduced activity. Weight loss 7.8%, moderate alopecia and hyperkeratosis, hunched posture, reduced activity.

RID, Rag2; IL-2rγ double KO NOD mice; RTKO, CD47; Rag2; IL-2rγ triple KO NOD mice; BSF, busulfan; TBI, total body irradiation; wpt, weeks post transplantation.

### Immune monitoring of the humanized mice

**Figure 2:**
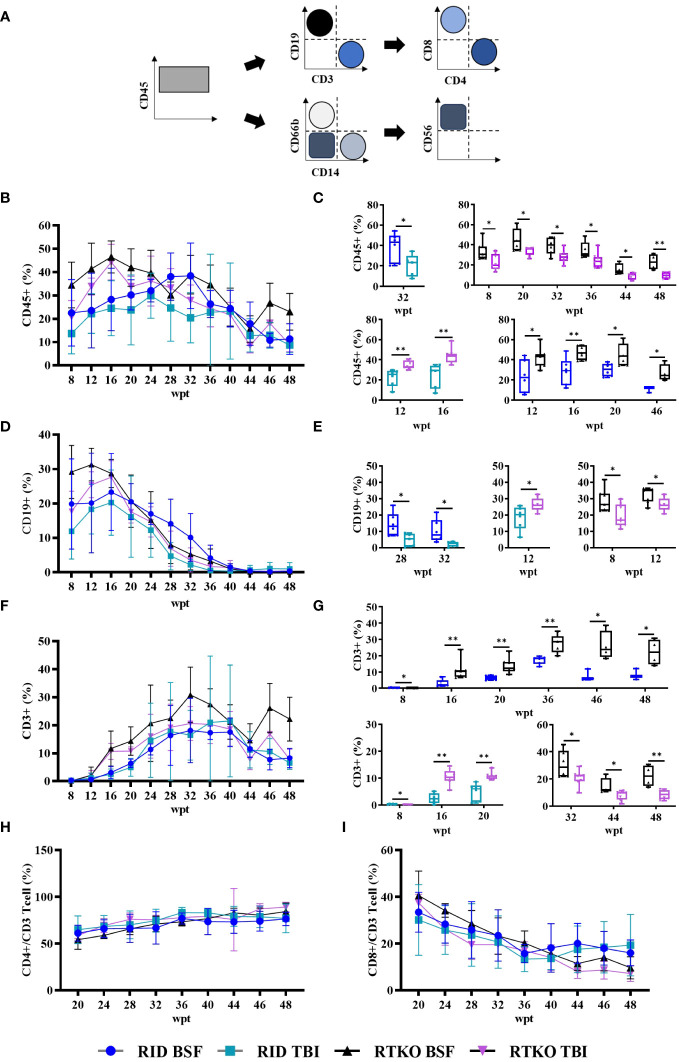
Immune monitoring of the humanized mouse. Engraftment of human cells was examined by FACS analysis from eight to 48 weeks after hCD34+ HSC injection. Sera were collected by retro orbital bleeding from mice. **(A)** Gating strategy for flow cytometry analysis. Levels and significant comparisons of **(B, C)** hCD45 leukocytes, **(D, E)** hCD19 B cells, **(F, G)** hCD3 T cells were examined. When hCD3+ cells were taken as a whole (100%), the percentages of **(H)** hCD4+ cells and **(I)** hCD8+ cells were calculated. Dot plots for significant comparisons of hCD45 (**Supplementary Figure S1**), hCD3 and hCD19 (**Supplementary Figure S2**), hCD4 and hCD8 (**Supplementary Figure S3**) were provided in supplementary figures. *p<0.05 and **p<0.01. h, human; FACS, fluorescence-activated cell sorting; RID, Rag2; IL-2rγ double KO NOD mice; RTKO, CD47; Rag2; IL-2rγ triple KO NOD mice; HSC, hematopoietic stem cell; BSF, busulfan; TBI, total body irradiation; wpt, weeks post transplantation.

To evaluate the human immune cells engraftment, flow cytometry for human leukocyte antigens was performed at intervals of four weeks or two weeks. After eight wpt, the hCD45 percentage of the DKO TBI group was the lowest (13.7%). The other groups had more than 20%, and that of the RTKO BSF group was the highest (34.5%). At most of the measurement points, the hCD45 percentages of the RTKO BSF group were the highest, and those of the DKO TBI group were the lowest. Finally, the hCD45 percentage of the RTKO BSF group was 23.0%, and in the other groups, it was around 10% at 48 wpt (**Figures 2B, C**). At the early stage of transplantation, most of the immune cells were hCD19+ B cells, but they gradually decreased to less than 2.0% from 40 wpt (**Figures 2D, E**). The percentage of hCD3+ T cells increased rapidly from 12 wpt, and they made up most of the human leukocytes after 40 wpt. At the beginning of the hCD3 T cells expansion, the percentage of hCD3+ T cells increased rapidly in the RTKO groups as compared to the DKO groups, and it was significantly greater at 16 and 20 wpt. Examining the results after 20 wpt, hCD4+ T cells among the hCD3+ T cells gradually increased from around 60.0% and finally increased to approximately 80.0%, whereas hCD8+ T cells continually decreased from about 40.0% to 10.0% (**Figures 2F–I**). Dot plots for significant comparisons of hCD45 (**Supplementary Figure S1**), hCD3 and hCD19 (**Supplementary Figure S2**), hCD4 and hCD8 (**Supplementary Figure S3**) were provided in the **Supplementary Material**. Other markers of leukocytes, including hCD14, hCD56, and hCD66b, were hardly detected, except for hCD66b in one mouse of the DKO BSF group (**Supplementary Figures S4–S6**).

### Pathological changes and assessment of the severity of GvHD

**Figure 3:**
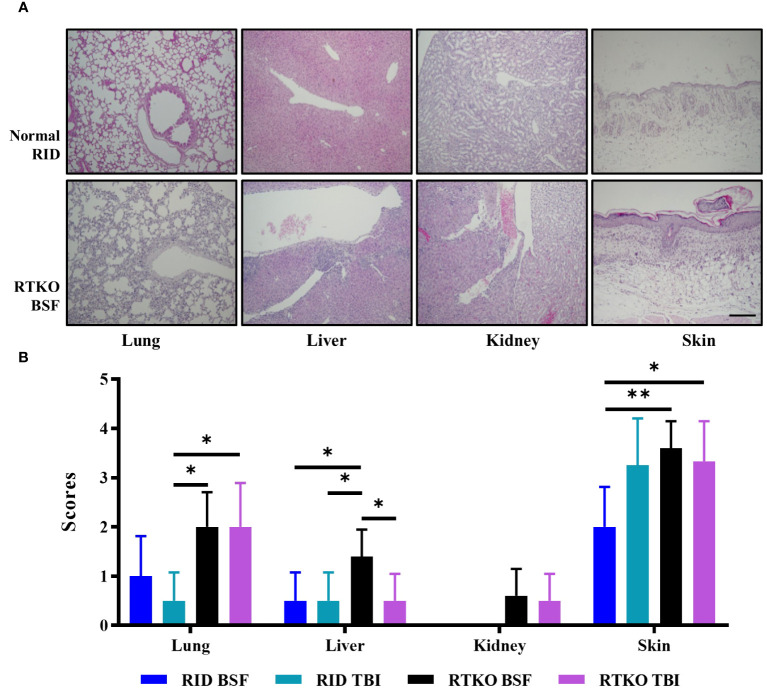
Histopathological findings and analyses of the semi-quantitative lesion scores. **(A)** Hematoxylin and eosin stains of lung, liver, kidney, and skin tissues of normal RID mice and the RTKO BSF group. Inflammatory cell aggregation was the major pathological change. Bar, 200 µm. **(B)** Semi-quantitative lesion scores of lung, liver, kidney and skin tissues of RID and RTKO groups. The scores of the RTKO BSF group were the highest in all organs. *p<0.05 and **p<0.01. RID, Rag2; IL-2rγ double KO NOD mice; RTKO, CD47; Rag2; IL-2rγ triple KO NOD mice; HSC, hematopoietic stem cell; BSF, busulfan; TBI, total body irradiation.

To determine the symptoms and severity of GvHD, histopathological analysis was performed. Inflammatory cell aggregation, mostly in the perivascular region, and some apoptotic cells were observed in the liver, lung, kidney, and skin. In the skin, epidermal hyperplasia and hyperkeratosis were prominent lesions (**Figure 3A**). Analyzing the semi-quantitative scoring system, pathological changes in the lung and liver were minimal to mild. Lung scores of the TKO groups were greater than the DKO BSF groups and significantly higher than the DKO TBI group. In the kidney, minimal inflammatory cell aggregation was observed only in some TKO group mice, and there were no significant differences among the groups. The skin lesions were the most severe, and the scores of the RTKO groups were significantly higher than the DKO TBI group. The scores of the RTKO BSF group were the highest in all organs; however, there were no life-threatening lesions (**Figure 3B**).

### Expression of human leukocyte antigens

**Figure 4:**
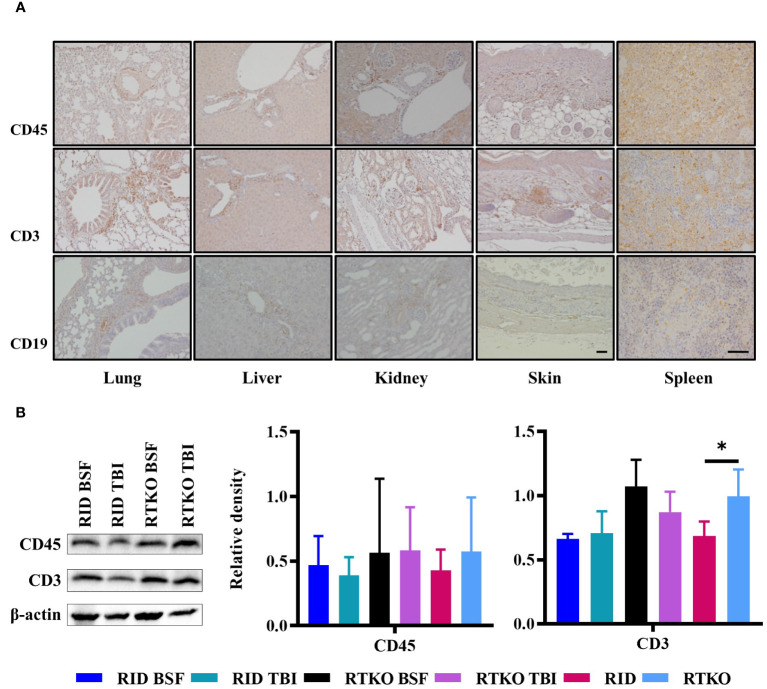
Expression of human leukocyte antigens. **(A)** Immunohistochemical staining of hCD45, hCD3, hCD19 in the lung, liver, kidney, skin, and spleen tissues of the RTKO groups. hCD45 and hCD3 were abundantly expressed in inflammatory cells in perivascular and subcutaneous areas, and some leukocytes were also stained with hCD19. Bar, 50 µm. **(B)** Western blot analysis of hCD45 and hCD3 proteins using the spleen tissues. Protein concentrations of the RTKO groups were greater than the RID groups, and the significant differences were only observed when comparing hCD3 results of all of the RTKO groups and the RID groups. *p<0.05. h, human; RID, Rag2; IL-2rγ double KO NOD mice; RTKO, CD47; Rag2; IL-2rγ triple KO NOD mice; HSC, hematopoietic stem cell; BSF, busulfan; TBI, total body irradiation.

Immunohistochemistry was performed to examine which types of human immune cells were infiltrating in affected tissues. Aggregating leukocytes in the perivascular area and interstitial tissues of the lung, liver, kidney, and spleen and subcutaneously infiltrating immune cells were stained with the hCD45 antibody. Most of the leukocytes were stained for hCD3 protein, and some immune cells expressed hCD19 (**Figure 4A**). To evaluate the engraftment and proliferation of the human leukocytes, the expression levels of hCD45, hCD3, and hCD19 proteins were analyzed using spleen tissues. hCD45 and hCD3 proteins were expressed more intensely in the RTKO groups than the RID groups, but there was no significant difference. Comparing the results of all of the RTKO groups and the RID groups, hCD3 was significantly higher in the RTKO group (**Figure 4B**). hCD19 was barely detected in all groups (data not shown).

### Measurement of human cytokine levels in mouse serum

**Figure 5:**
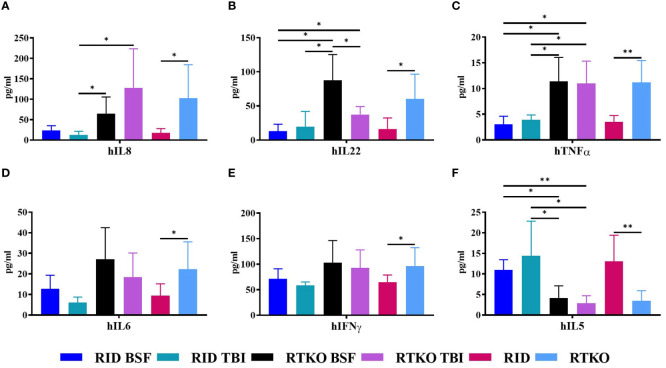
Measurement of human cytokine concentrations in mouse serum. The levels of human cytokines were analyzed using Multiplex ELISA. The sera were collected after sacrifice, followed by assessment of the amounts of **(A)** hIL-8, **(B)** hIL-22, **(C)** hTNFα, **(D)** hIL-6, **(E)** hIFNγ and **(F)** hIL-5. In the RTKO group, the expression of hIL-6, hIL-8, hIL-22, hTNFα, and hIFNγ increased, whereas the expression of hIL-5 decreased. *p<0.05 and **p<0.01. h, human; RID, Rag2; IL-2rγ double KO NOD mice; RTKO, CD47; Rag2; IL-2rγ triple KO NOD mice; BSF, busulfan; TBI, total body irradiation; IL, Interleukin; IFNγ, Interferon Gamma; TNFα, tumor necrosis factor alpha.

Human cytokine concentrations were measured to elucidate the cause of GvHD associated with the inflammatory cell aggregation using Human CorPlex Cytokine Panel 1 10-Plex Array (Quanterix). Serum concentrations of hIL-8, hIL-22, and hTNFα were significantly increased in both RTKO groups relative to the RID groups (**Figures 5A–C**). In addition, the concentrations of hIL-6 and hIFNγ were enhanced in the RTKO groups; however, significant differences were only obtained when comparing all of the RTKO groups and the RID groups (**Figures 5D, E**). On the other hand, the levels of hIL-5 were significantly decreased in the RTKO groups (**Figure 5F**). There were no meaningful differences in the other cytokines (IL-1β, IL-4, IL-10, and IL-12p70; data not shown).

## Discussion

In this study, we generated hCD34+ humanized mice using RID and RTKO mice, and two pre-conditioning methods, TBI and BSF injection, were compared. The production of humanized mice is generally determined as successful if the hCD45 percentage in mouse PBMC is more than 25% (3). Considering this standard and the health of our mice, including the body weights and survival rates (**Figures 1C, D**) and histopathological analysis (**Figure 3**), humanized mice were successfully generated in all groups. At 8 wpt, the hCD45 percentage of the RTKO BSF was more than 30%, and in the other groups, it was subsequently over 25%. The engraftment of human leukocytes was maintained for a long period of time. Greater than 25% of the hCD45 percentage was maintained in most groups until 40 wpt, and those of the RID TBI group with the lowest results were more than 20% during the same time frame (**Figure 2B**). After 40 wpt, the ratio of hCD45 decreased (**Figure 2B**) and the fatalities increased (**Figure 1D**). Therefore it was decided to finish the experiment at 48 wpt. However, it is predicted that the experimental window of the RTKO BSF group could be extended beyond 48 wpt, as the ratio of hCD45 was maintained at 23.0% at the last measurement in that group (**Figure 2B**).

Considering the body weight ratio and the histological evaluation, the RID BSF group had the best health condition, and the RTKO BSF group, which had the highest histopathological scores, was also healthy, with a body weight increase of 156.5% by the end of the experiment (**Figures 1C**, **3B**). The final survival rate at 48 wpt was the lowest in the RID BSF group (50.0%), and the highest in the RTKO TBI group (71.4%), with no significant difference among the groups (**Figure 1D**). Although mortality was observed in all groups until 48 wpt, only two animals died before 40 wpt in all groups (one mouse in each of the RID TBI and the RTKO BSF groups) (**Figure 1D**). And there were no major health problems other than skin lesions except for mortalities (**Figure 3**). Considering the animal health and human leukocytes engraftment in this study, as well as the experimental period in other studies (21–24), the humanized mouse models of all applied conditions in this study is expected to be suitable models for long-term experiments.

Human leukocytes reconstructed *in vivo* were mostly hCD19 + B cells at the beginning, but they gradually decreased to less than 2% after 40 wpt. The percentage of hCD19 + B cells in the RTKO BSF group was the highest until 20 wpt, and after that, it was the highest in the RID BSF group until 40 wpt (**Figure 2D**). These results were attributed to the earlier differentiation of human leukocytes in RTKO mice than RID mice, resulting in an increase in hCD3+ T cells. The percentage of hCD3+ T cells increased from 12 wpt. Human leukocytes were differentiated more rapidly in the RTKO groups, and the percentage of hCD3 cells exceeded 10% at 16 wpt, which was eight weeks earlier than those of the RID groups (**Figure 2F**). After 16 wpt, hCD3+ T cells were higher in the RTKO BSF group, and the percentage of hCD4 to hCD8 was also the highest in the RTKO BSF group at almost all measurement points (**Figures 2H, I**). In addition, the human immune cells in the spleen were significantly engrafted in the RTKO groups (**Figure 4B**). Interpreting the results of leukocytes engraftment and differentiation, the RTKO mouse is a more suitable platform than the RID mouse, and BSF injection is a more appropriate pre-conditioning method than TBI for generating the hCD34+ humanized mice.

In histopathological analysis, GvHD lesions were prominent in the skin, not severe in the lungs and liver (**Figure 3**), and there were no life-threatening lesions except in the dead mice (**Figure 1D**). Most of the immune cells aggregated in the tissues were hCD3+ T cells (**Figure 4A**). The expression of hIL-6, hIL-8, hIL-22, hTNFα, and hIFNγ was increased in the RTKO mice (**Figure 5**). Associated with T lymphocytes, IFNγ and TNFα are core cytokines of chronic GvHD pathogenesis (25), and IL-6 and IL-22 aggravate skin lesions (26). IL-8 has a function of lymphocyte recruitment (27). These results suggest that cytokines secreted by the human T cells infiltrating tissues played a crucial role in the development of GvHD lesions and this mainly affecting skin pathogenesis. On the other hand, hIL-5 was decreased in RTKO mice, as hIL-5 is decreased in a CD47 blockade (28). hIL-5, classified as a Th2-type cytokine (29), is known to be involved in both acute and chronic GvHD pathogenesis, but the exact mechanism is not yet clearly understood (30, 31). hIL-5 is produced by CD4 T cells (32) and the enhancement of hIL-8 could reduce the activation of CD4 T cells (33). So, it is interpreted that the increased levels of hIL-8 (**Figure 5A**) in the RTKO groups may reduce the activation of CD4 T cells, thereby reducing the secretion of hIL-5 from CD4 T cells. We also hypothesize that the intensity of GvHD might be alleviated due to the reduction of hIL5 in RTKO, resulting in no significant differences in the survival rate among groups.

Recently, mice modified with the CD47-SIRPA axis have been studied to improve humanized mouse models (13, 14, 34). This is because the CD47 deficient condition could enhance tolerance for transplanted human leukocytes (35). However, due to the strong affinity between human SIRPA and mouse CD47 (8), such trials have been rarely applied in NOD mice. In this study, hCD34+ humanized mice were generated using CD47 KO NOD mice for the first time, and the CD47 deficiency enhanced human immune cell engraftment (**Figures 2**, **4B**), like in studies using the C57BL/6 strain (13, 14). Compared to humanized mice studies applying NSG mice, it was also confirmed that the engraftment and the differentiation of human immune cells were enhanced in RTKO mice (3, 23, 36, 37). Considering the improvement in reproducing human immune system and the additional benefits of rag2 gene mutation, including irradiation tolerance and the deficiency of T/B cell leakiness (8, 9), RTKO mice could be an alternative platform for generating humanized mice.

There are two errors in this study. The first is an error in the FACS analysis. The human leukocyte antigen analysis in the RTKO groups was underestimated at 44 wpt. When checking the results at that time, we judged that it was a simple decrease, but considering the high values at 40, 46, and 48 wpt, we expected an underestimation of the results due to experimental error. Therefore, it is possible that the RTKO BSF group maintained a more stable human leukocytes engraftment until the end of the experiment. The second error is the death of mice due to breeding management faults. On the weekend of 44 wpt, two mice in the RID BSF group died in the same cage due to water supply problems (**Table 2**). Considering the weight change (**Figure 1C**) and the clinical signs of these mice, they were expected to have survived, and if so, the RID BSF group might have had the highest survival rate (83.3%, n=6), and might have been the most stable model to maintain a good health condition.

In conclusion, CD34+ humanized mouse models were successfully established in all four groups using RID and RTKO mice with two pre-conditioning methods, TBI and BSF. Among them, the RTKO BSF group was identified as the most suitable model considering the improvement of human leukocytes reconstruction and the extended experimental window beyond 48 wpt. This model is expected to be a novel and useful platform for various immune research, including cancer immunotherapy, virology, hematology, and autoimmunity. However, further studies are needed reduce the mortality and enhance the expansion of immune cells other than B and T cells.

## Data availability statement

The original contributions presented in the study are included in the article/**Supplementary Material**. Further inquiries can be directed to the corresponding author.

## Ethics statement

Ethical approval was not required for the studies on humans in accordance with the local legislation and institutional requirements because only commercially available established cell lines were used. The animal study was approved by The Institutional Animal Care and Use Committee of Asan Institute for Life Sciences (Seoul, Korea, IACUC No. 2020-12-093). The study was conducted in accordance with the local legislation and institutional requirements.

## Author contributions

KK: Formal analysis, Writing – original draft, Validation, Investigation. SL: Conceptualization, Supervision, Writing – review & editing. IB: Supervision, Writing – review & editing, Resources. HS: Formal analysis, Validation, Writing – original draft, Investigation. SJ: Formal analysis, Investigation, Writing – original draft. JR: Formal analysis, Investigation, Methodology, Writing – original draft. SR: Formal analysis, Investigation, Methodology, Writing – original draft. JS: Conceptualization, Writing – review & editing, Methodology, Validation. JK: Conceptualization, Supervision, Validation, Writing – review & editing. SH: Conceptualization, Supervision, Validation, Writing – original draft, Writing – review & editing.
