# Supplementary material for: CD47;Rag2;IL-2rγ triple knock-out mice pre-conditioning with busulfan could be a novel platform for generating hematopoietic stem cells engrafted humanized mice
Source: Front Immunol. 2024 Jul 26;15:1365946. doi: 10.3389/fimmu.2024.1365946 (PMC11310007; doi:10.3389/fimmu.2024.1365946)
Supplement: Supplementary file 1 [file DataSheet_1.docx]

***Supplementary Material***

**CD47;Rag2;IL-2rγ triple knock-out mice pre-conditioning with busulfan could be a novel platform for generating hematopoietic stem cells engrafted humanized mice**

Kang-Hyun KIM^1,2^, Sang-wook Lee^3^, In-Jeoung Baek^1,4^, Hye-Young Song^4^, Seon-Ju Jo^4^, Je-Won Ryu^1^, Seung-Hee Ryu^4^, Jin-Hee Seo^5^, Jong-Choon Kim^2†^ and Seung-Ho Heo^1,4*†^

^1^Convergence Medicine Research Center, Asan Medical Center, Seoul, Republic of Korea

^2^College of Veterinary Medicine, Chonnam National University, Gwangju, Republic of Korea

^3^Department of Radiation Oncology, Asan Medical Center, Seoul, Republic of Korea

^4^Asan Institute for Lifesciences, Asan Medical Center, Seoul, Republic of Korea

^5^Laboratory Animal Center, Korea Institute of Radiological and Medical Sciences, Seoul, Republic of Korea

**Correspondence:** Seung-Ho Heo^*^ [bd0226@amc.seoul.kr](mailto:bd0226@amc.seoul.kr)

## Supplementary Figures

**
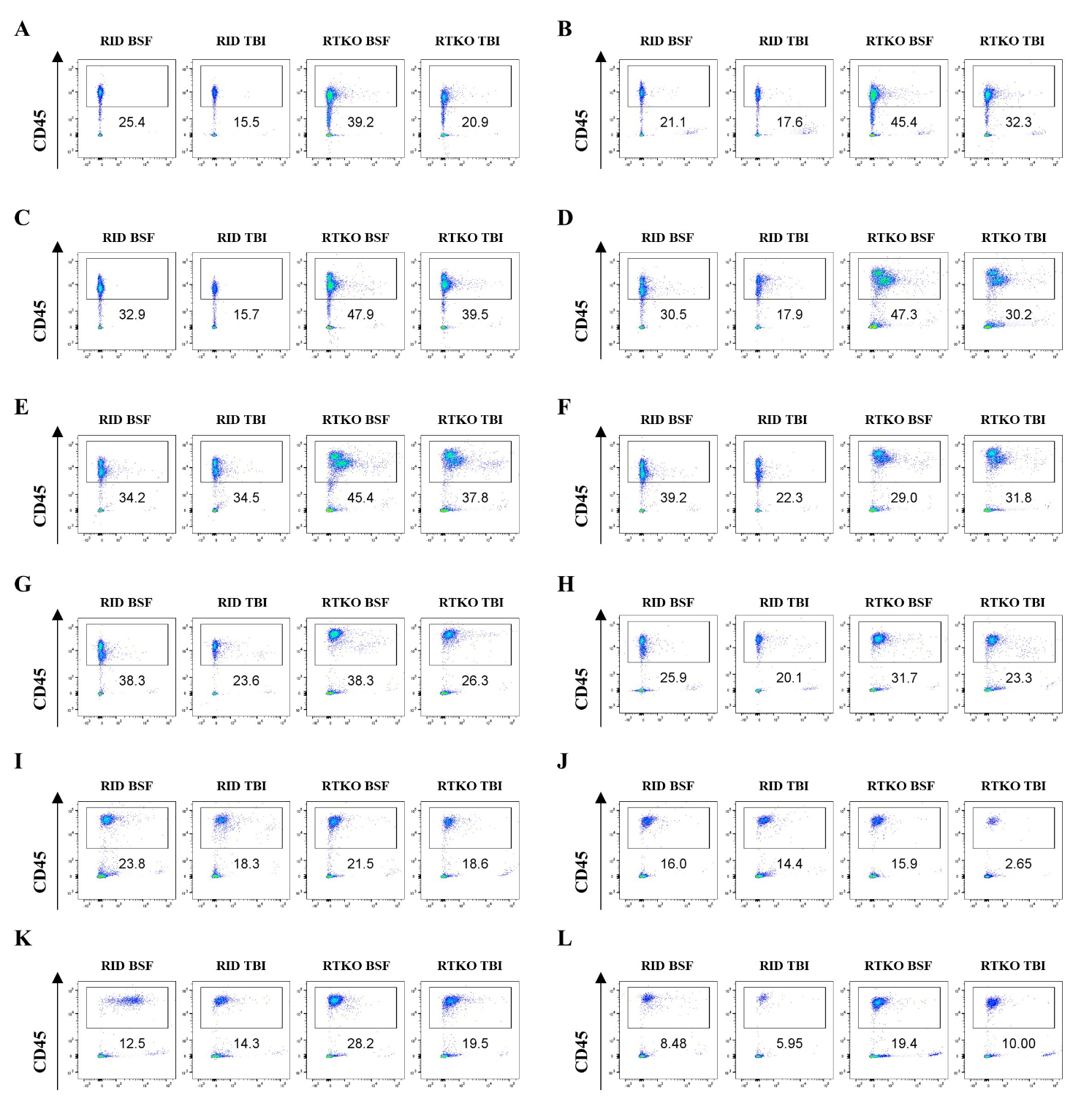
**

**Supplementary Figure 1.** Immune monitoring of the humanized mouse. Engraftment of human cells was examined by FACS analysis. Representative dot plots of hCD45 at (A) 8 weeks, (B) 12 weeks, (C) 16 weeks, (D) 20 weeks, (E) 24 weeks, (F) 28 weeks, (G) 32 weeks, (H) 36 weeks, (I) 40 weeks, (J) 44 weeks, (K) 46 weeks, and (L) 48 weeks after hCD34+ HSC injection; FACS, fluorescence-activated cell sorting; RID, Rag2; IL-2rγ double KO NOD mice; RTKO, CD47; Rag2; IL-2rγ triple KO NOD mice; HSC, hematopoietic stem cell; BSF, busulfan; TBI, total body irradiation

**
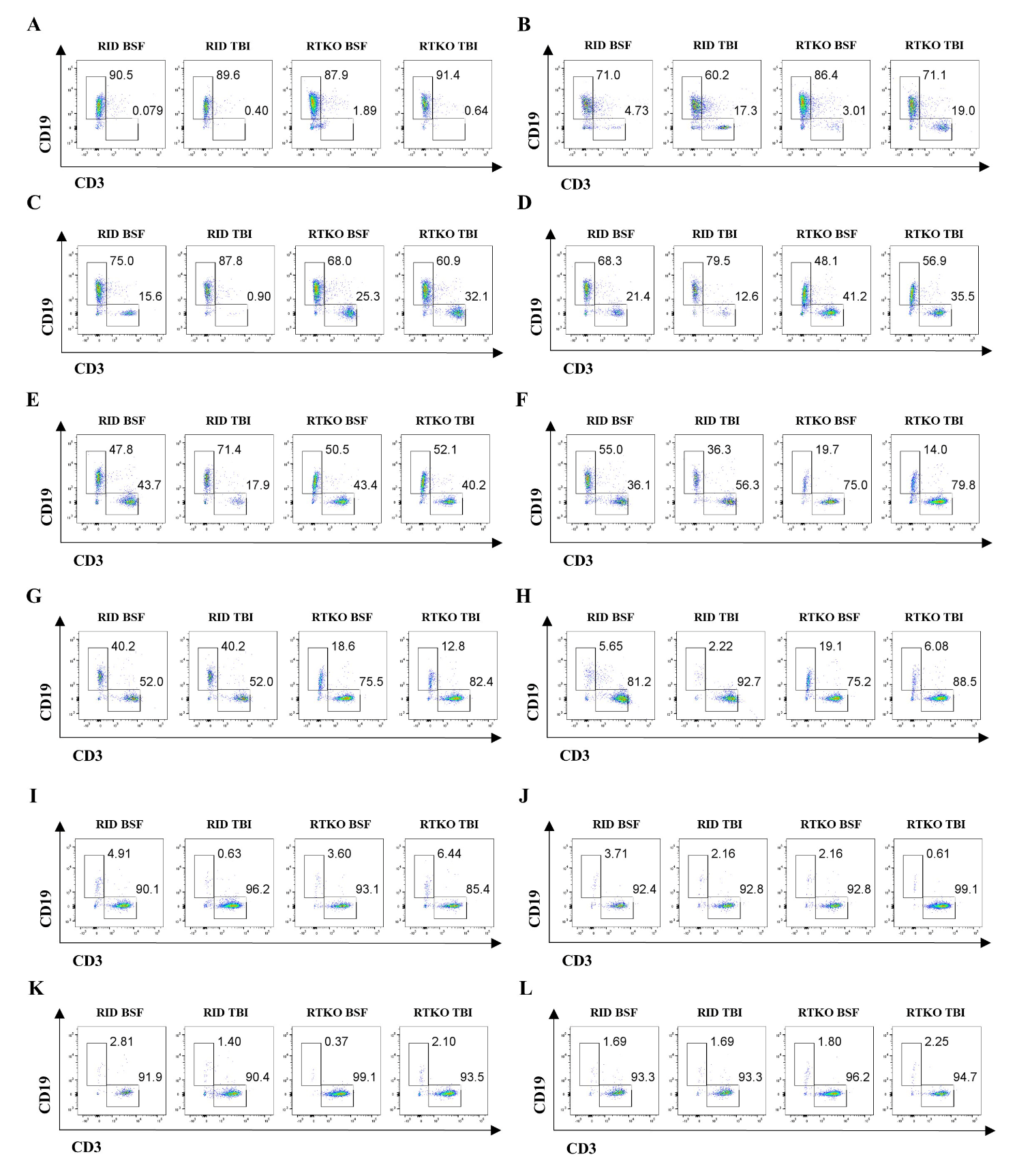
**

**Supplementary Figure 2.** Immune monitoring of the humanized mouse. Engraftment of human cells was examined by FACS analysis. Representative dot plots of hCD3 and hCD19 at (A) 8 weeks, (B) 12 weeks, (C) 16 weeks, (D) 20 weeks, (E) 24 weeks, (F) 28 weeks, (G) 32 weeks, (H) 36 weeks, (I) 40 weeks, (J) 44 weeks, (K) 46 weeks, and (L) 48 weeks after hCD34+ HSC injection; FACS, fluorescence-activated cell sorting; RID, Rag2; IL-2rγ double KO NOD mice; RTKO, CD47; Rag2; IL-2rγ triple KO NOD mice; HSC, hematopoietic stem cell; BSF, busulfan; TBI, total body irradiation.

**
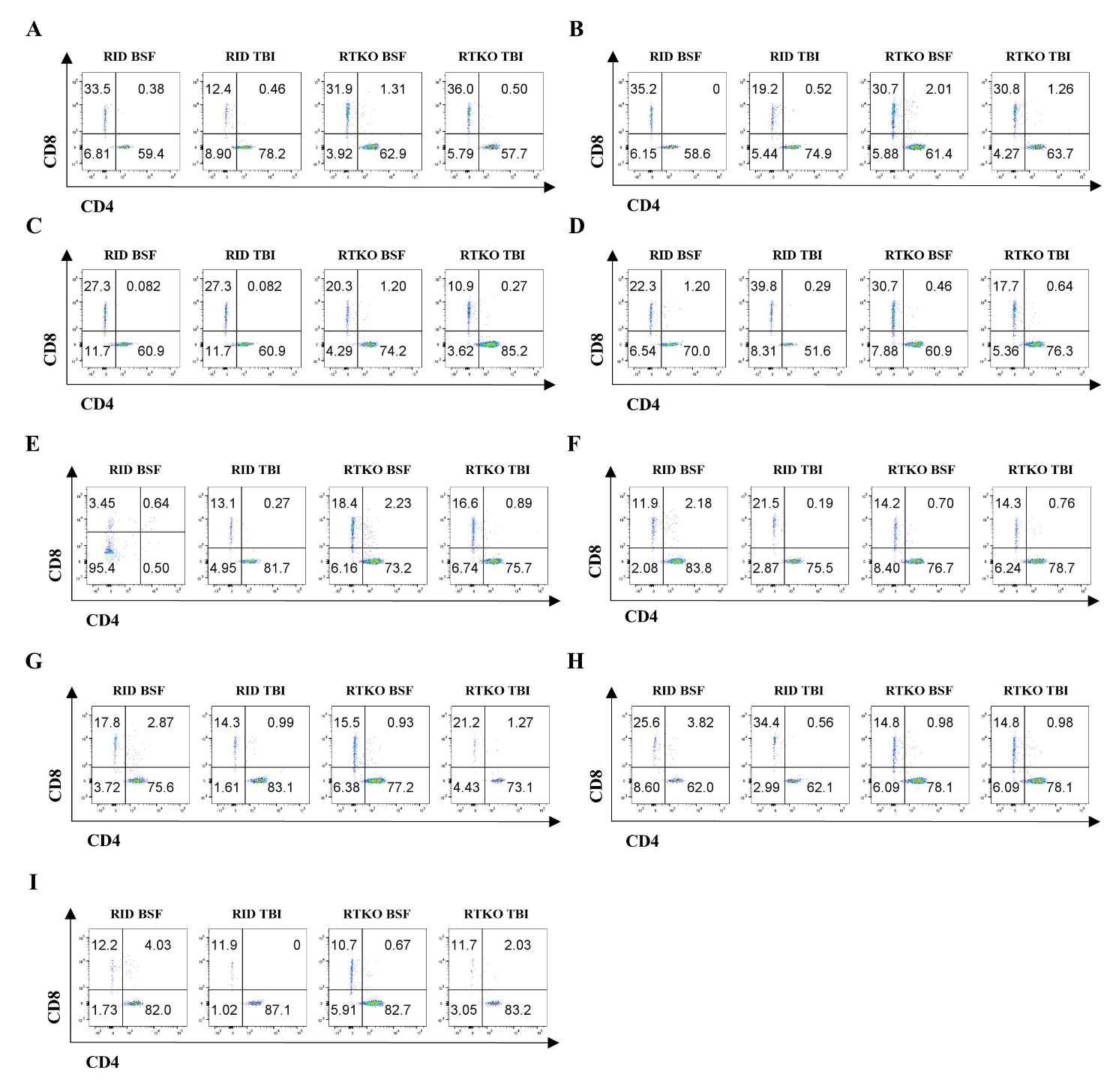
**

**Supplementary Figure 3.** Immune monitoring of the humanized mouse. Engraftment of human cells was examined by FACS analysis. Representative dot plots of hCD4 and hCD8 at (A) 20 weeks, (B) 24 weeks, (C) 28 weeks, (D) 32 weeks, (E) 36 weeks, (F) 40 weeks, (G) 44 weeks, (H) 46 weeks, and (I) 48 weeks after hCD34+ HSC injection; FACS, fluorescence-activated cell sorting; RID, Rag2; IL-2rγ double KO NOD mice; RTKO, CD47; Rag2; IL-2rγ triple KO NOD mice; HSC, hematopoietic stem cell; BSF, busulfan; TBI, total body irradiation


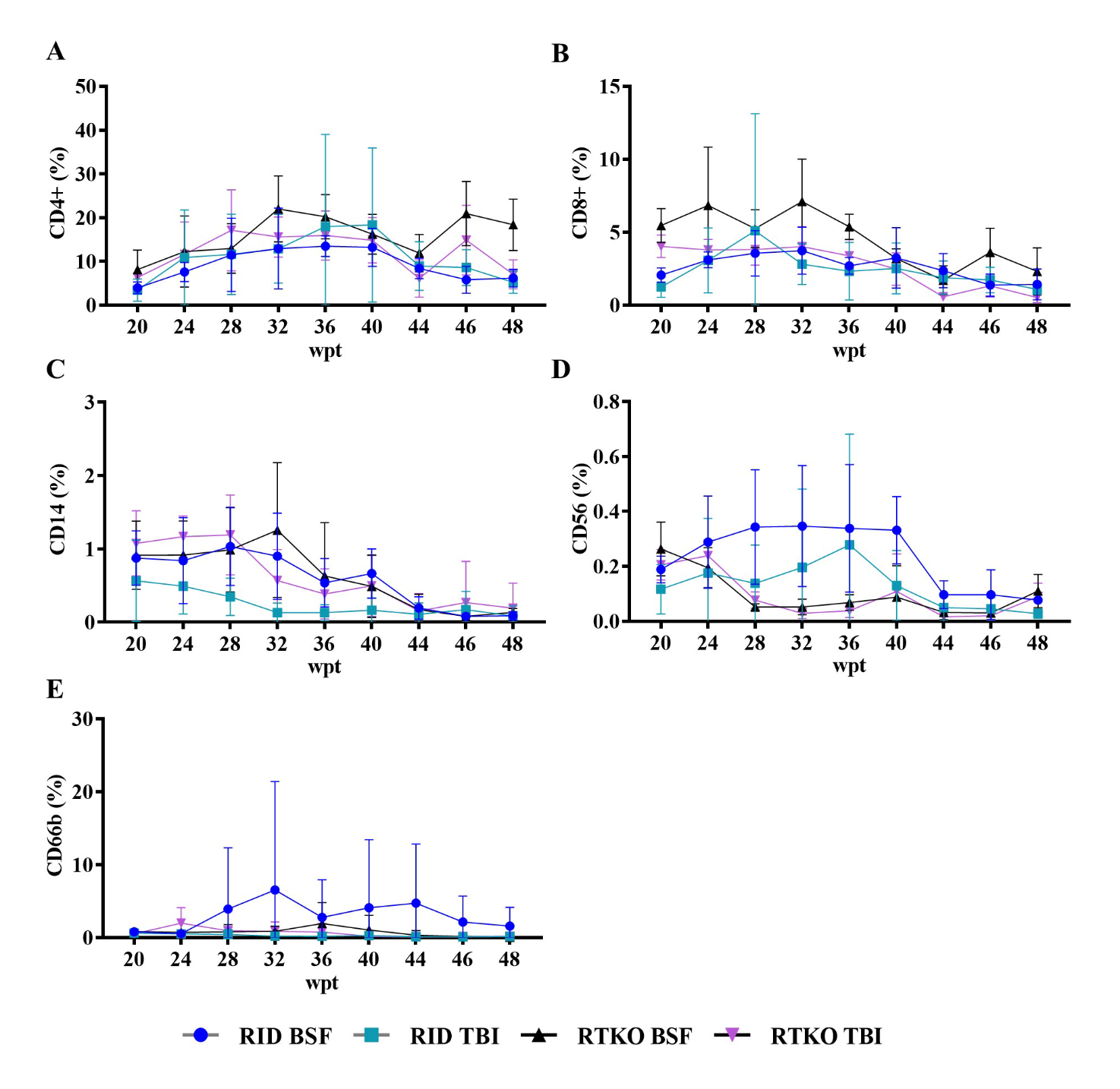


**Supplementary Figure 4.** Immune monitoring of the humanized mouse. Engraftment of human cells was examined by FACS analysis from 20 to 48 weeks after hCD34+ HSC injection. The sera were collected by retro orbital bleeding from mice, and levels of (A) hCD4+, (B) hCD8+, (C) hCD14+, (D) hCD56+, and (E) hCD66b+ cells were evaluated. h, human; FACS, fluorescence-activated cell sorting; RID, Rag2; IL-2rγ double KO NOD mice; RTKO, CD47; Rag2; IL-2rγ triple KO NOD mice; HSC, hematopoietic stem cell; BSF, busulfan; TBI, total body irradiation.


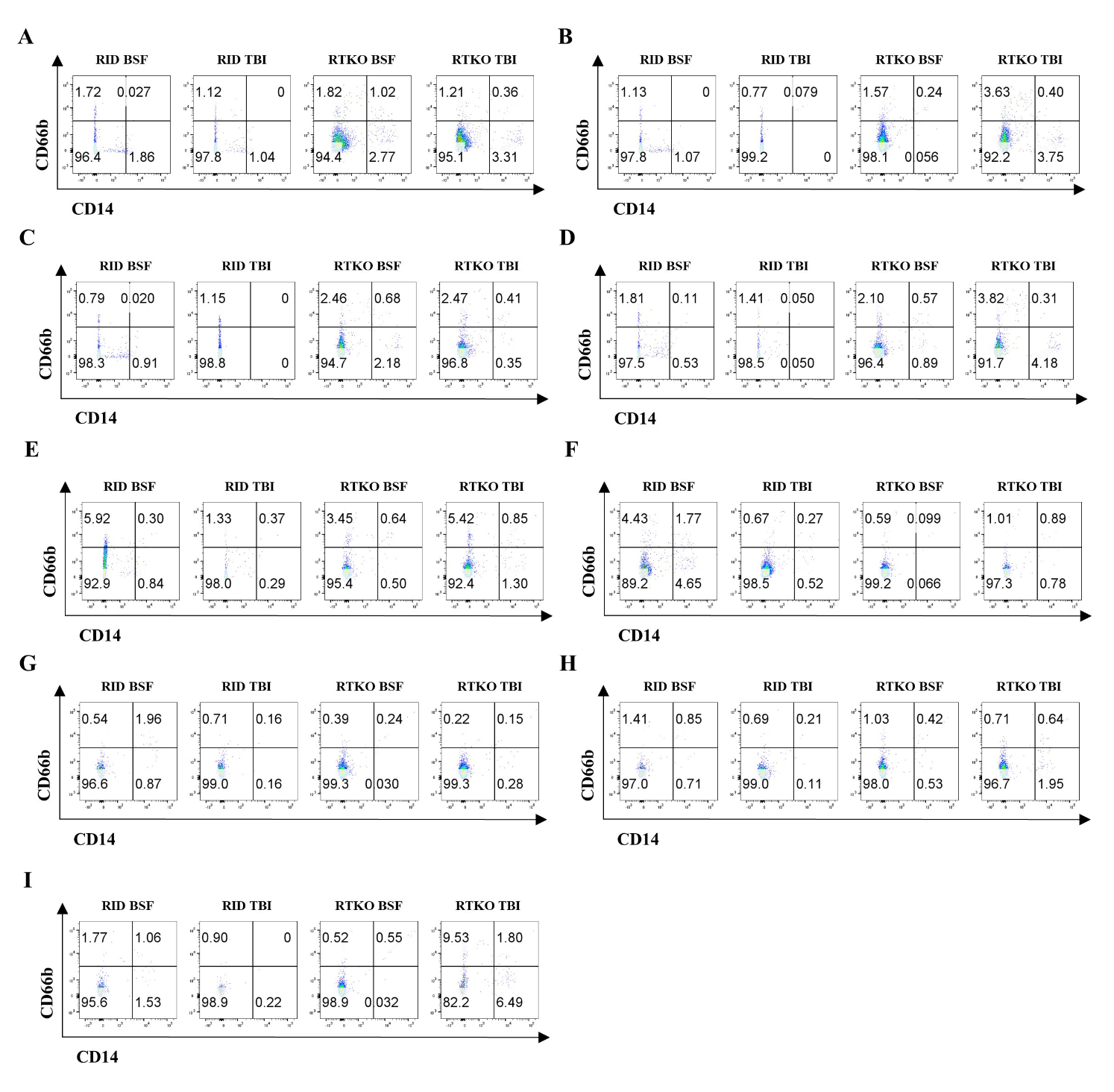


**Supplementary Figure 5.** Immune monitoring of the humanized mouse. Engraftment of human cells was examined by FACS analysis. Representative dot plots of hCD14 and hCD66b at (A) 20 weeks, (B) 24 weeks, (C) 28 weeks, (D) 32 weeks, (E) 36 weeks, (F) 40 weeks, (G) 44 weeks, (H) 46 weeks, and (I) 48 weeks after hCD34+ HSC injection; FACS, fluorescence-activated cell sorting; RID, Rag2; IL-2rγ double KO NOD mice; RTKO, CD47; Rag2; IL-2rγ triple KO NOD mice; HSC, hematopoietic stem cell; BSF, busulfan; TBI, total body irradiation


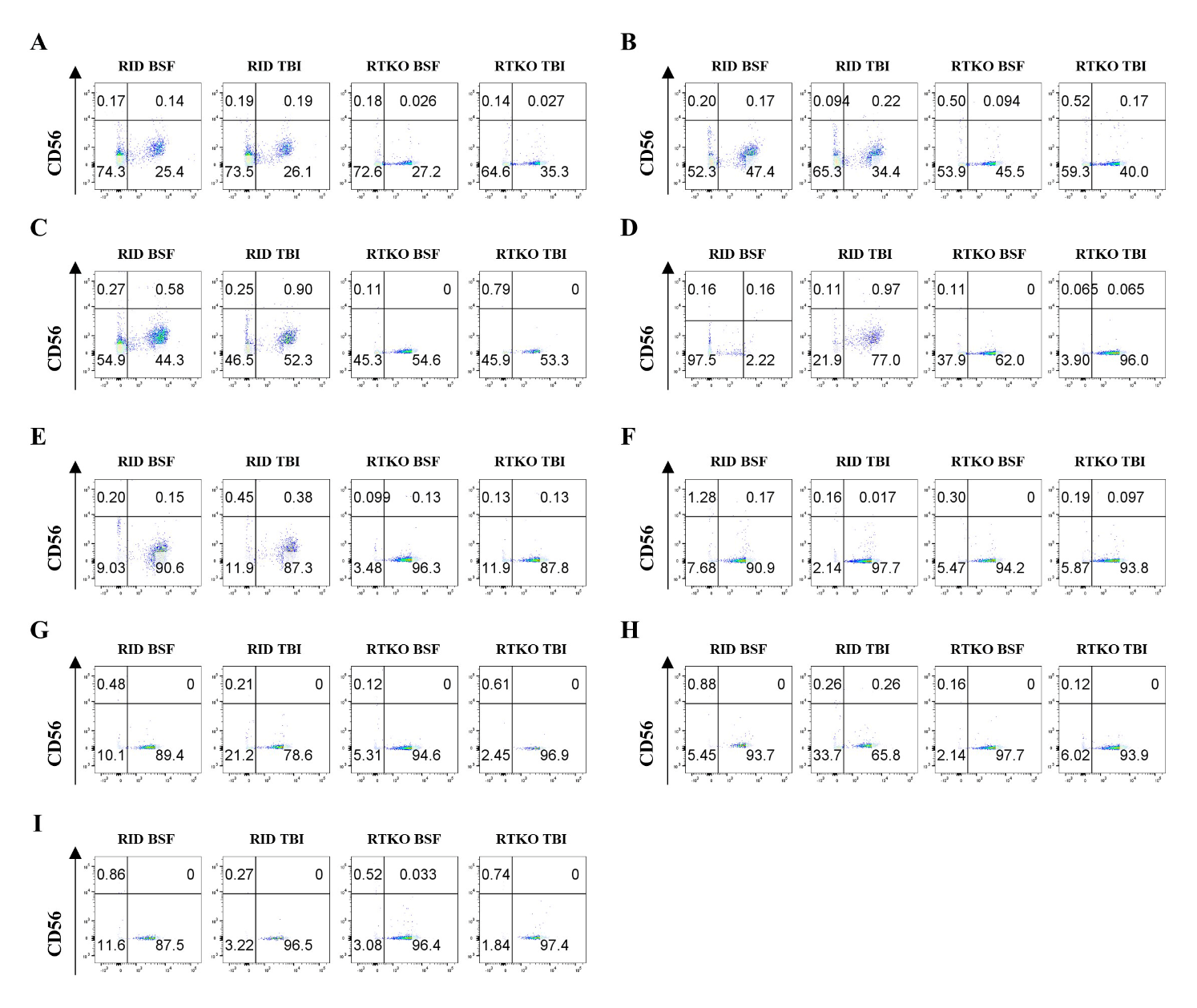


**Supplementary Figure 6.** Immune monitoring of the humanized mouse. Engraftment of human cells was examined by FACS analysis. Representative dot plots of hCD56 at (A) 20 weeks, (B) 24 weeks, (C) 28 weeks, (D) 32 weeks, (E) 36 weeks, (F) 40 weeks, (G) 44 weeks, (H) 46 weeks, and (I) 48 weeks after hCD34+ HSC injection; FACS, fluorescence-activated cell sorting; RID, Rag2; IL-2rγ double KO NOD mice; RTKO, CD47; Rag2; IL-2rγ triple KO NOD mice; HSC, hematopoietic stem cell; BSF, busulfan; TBI, total body irradiation
